# Excess Mortality Is Not Synonymous with COVID-19–Related Deaths

**DOI:** 10.4269/ajtmh.22-0243a

**Published:** 2022-06-13

**Authors:** Camilla Mattiuzzi, Giuseppe Lippi

**Affiliations:** Service of Clinical Governance Provincial Agency for Social and Sanitary Services Trento, Italy E-mail: camilla.mattiuzzi@apss.tn.it; Section of Clinical Biochemistry and School of Medicine University of Verona Verona, Italy E-mail: giuseppe.lippi@univr.it

Dear Sir,

We read with interest the article by Leffler et al.,^1^ who attempted to provide an estimation of COVID-19–related mortality in India in terms of excess mortality compared with the pre-pandemic years 2015–2019. We would like to raise some concerns about the assumption that excess mortality directly translates into COVID-19–related deaths. The first important aspect to be considered is that many countries, including India, have established strict restrictive measures to limit virus spread that have limited personal freedom (i.e., prohibition of mass gatherings or public events, lockdowns, and curfews).^2^ Therefore, limitation in the circulation of people has contributed to a remarkably reduced burden of road injuries and other unintentional outdoor accidents, which are among the most frequent causes of death in the general population.^3^ The adoption of many restrictive measures has also generated unfavorable consequences for the clinical management of a kaleidoscope of acute and chronic pathologies (e.g., acute myocardial infarction, cancer, and diabetes). Accordingly, it has been estimated that the lack of or delayed care throughout the COVID-19 pandemic may have affected more than 10% of emergency practices and more than 30% of routine clinical services, thus contributing to an excess of otherwise avoidable deaths not directly attributed to COVID-19.^4^ For these reasons, we do not agree with the syllogism that excess mortality during the pandemic directly indicates COVID-19–related mortality.
